# Green Space and Child Weight Status: Does Outcome Measurement Matter? Evidence from an Australian Longitudinal Study

**DOI:** 10.1155/2015/194838

**Published:** 2015-09-02

**Authors:** Taren Sanders, Xiaoqi Feng, Paul P. Fahey, Chris Lonsdale, Thomas Astell-Burt

**Affiliations:** ^1^School of Science and Health, University of Western Sydney, Penrith, NSW 2751, Australia; ^2^School of Health and Society, University of Wollongong, Wollongong, NSW 2722, Australia; ^3^Menzies Centre for Health Policy, University of Sydney, Sydney, NSW 2006, Australia; ^4^Boden Institute of Obesity, Nutrition, Exercise and Eating Disorders, University of Sydney, Sydney, NSW 2006, Australia; ^5^Institute for Positive Psychology and Education, Australian Catholic University, Strathfield, NSW 2135, Australia; ^6^School of Geography and Geosciences, University of St Andrews, North Street, St Andrews KY16 9AL, UK

## Abstract

*Objective*. To examine whether neighbourhood green space is beneficially associated with (i) waist circumference (WC) and (ii) waist-to-height ratio (WtHR) across childhood. *Methods*. Gender-stratified multilevel linear regressions were used to examine associations between green space and objective measures of weight status in the Longitudinal Study of Australian Children, a nationally representative source of data on 4,423 children aged 6 y to 13 y. WC and WtHR were measured objectively. Percentage green space within the local area of residence was calculated. Effect modification by age was explored, adjusting for socioeconomic confounding. *Results*. Compared to peers with 0–5% green space locally, boys and girls with >40% green space tended to have lower WC (*β*_boys_  −1.15, 95% CI −2.44, 0.14; *β*_girls_  −0.21, 95% CI −1.47, 1.05) and WtHR (*β*_boys_  −0.82, 95% CI −1.65, 0.01; *β*_girls_  −0.32, 95% CI −1.13, 0.49). Associations among boys were contingent upon age (*p*  values_age*∗*green  space_ < 0.001) and robust to adjustment for socioeconomic variables. The benefits of greener neighbourhoods appeared from age 7, with mean WC and WtHR for boys aged 13 y with >40% green space at 73.85 cm and 45.75% compared to those with 0–5% green space at 75.18 cm and 46.62%, respectively. *Conclusions*. Greener neighbourhoods appear beneficial to alternative child weight status measures, particularly among boys.

## 1. Introduction

Previous research has found longitudinal associations between children's proximity to green space (e.g., parks) and their body mass index (BMI) [1]. Generally, it has been found that children living in areas comprised of moderate amounts of green space (i.e., 6–30%) had lower BMIs than those with little to no green space (i.e., 0–5%) after adjusting for socioeconomic differences, with older boys having the strongest association. It is unclear, however, if the influence of green space is consistent across different measures of weight status.

Weight status is routinely used to assess children's risk for health complications and cardiovascular disease in later life. While BMI is the most widely used measure, evidence suggests that waist circumference (WC) and waist-to-height ratio (WtHR) may be better predictors of cardiovascular disease in children [2]. Inaccuracies in risk estimation derived from BMI are likely due to differences in body composition (i.e., levels of body fat) [3]. Both WC and WtHR are more likely to take into account levels of visceral fat than BMI [4].

To the authors' knowledge, no previous study has examined the role of green space on children's WC or WtHR, with previous research relying on either objectively measured (e.g., [5]) or self-reported (e.g., [6]) BMI. It therefore remains unclear how green space may influence measures which are more sensitive to changes in body fat. Therefore, the purpose of this study was to examine the longitudinal influence of green space on waist circumference and waist-to-height ratio.

## 2. Methods

### 2.1. Data

Data for this study comes from the Longitudinal Study of Australian Children (LSAC). The full methodology of the LSAC is described elsewhere [7]. Briefly, the LSAC is a large-scale project funded by the Australian Government. A two-stage clustered design was used, with eligible children identified through the Medicare database, Australia's universal health care service. The postcodes in which these children lived were then stratified by state and urban or rural status and a sample of postcodes chosen to comprise the sample. Children were initially 4-5 years old at the first data collection period (*n* = 4983). As body composition is inconsistent before age 5 [8], we used data from waves 2–5 only.

Two measures of weight status were the outcome variables for the present study: WC and WtHR. Weight status was measured biennially during face-to-face interviews. The interviewer measured the child's height and WC to the nearest 0.1 cm using a portable stadiometer (Invicta, Code IPO955) and a tape measure. Two measurements were taken and the average used. When the difference between the measures was >0.5 cm, a third measurement was taken and the average of the two closest measures was used [9]. The child's WtHR was determined by dividing waist circumference (cm) by height (cm) and multiplied by 100 to represent WC as a percentage of height.

The proportion of neighbourhood green space was calculated for each statistical area level 2 (SA2), the smallest area measure available in the LSAC [10] with an average population of 10,000 individuals per area. Mesh blocks [11] from 2006, which classify small-scale areas based on the main land use, were provided by the Australian Bureau of Statistics. To calculate green space, we included all mesh blocks which were classified as parkland, excluding those reserved for agricultural use [12]. Each SA2 was then assigned to a proportion of green space. Due to generally low proportions of green space in areas, we grouped percentage green space into the following categories: 0–5%, 6–10%, 11–20%, 21–30%, 31–40%, and >40%.

As socioeconomic and demographic status are likely confounders in the relationship between green space and weight status [13], measures of socioeconomic status were included as controls. The total combined weekly income of caregivers, the child's indigenous status (i.e., Australian Aboriginal or Torres Strait Islander) [14], if the child spoke a language other than English at home, and the number of years of education the mother had received on a scale ranging from 0 (i.e., never attended school) to 20 (completed postgraduate degree) [15] were included as repeated measures in the fully adjusted model.

### 2.2. Statistical Analysis

Multilevel linear regression was used to model the influence of neighbourhood green space on difference measures of weight status, to account for clustering within neighbourhoods. A “null” model was fit first, consisting of only the outcome measure and the following hierarchical structure: the measure of weight status at each time point (level 1), nested within individuals (level 2), and nested within Statistical Areas Level 2s (level 3). Polynomial functions of age were tested for each outcome measure and included only where they significantly improved model fit. Green space was then added (model 1), followed by a green space by age interaction (model 2). Finally, an adjusted model was fit, controlling for socioeconomic status (model 3). Log-likelihood tests were used to determine if explanatory variables significantly impacted on the models, with significance levels set at 5%. Multilevel model growth curves were generated from the final models to further investigate the associations. To investigate gender differences in weight status trajectories, we chose to fit gender-stratified models for all outcomes. All statistical analysis was conducted using STATA 12 (StataCorp, College Station, TX, USA).

## 3. Results

The sample is described in Table 1. At the area level (i.e., level 3) an unadjusted intraclass correlation (ICC) of 0.03 was found for boys' WCs and 0.04 for girls' WCs. The correlation was similar for WtHR, with an unadjusted ICC of 0.06 for boys and 0.04 for girls.

**Figure 1:**
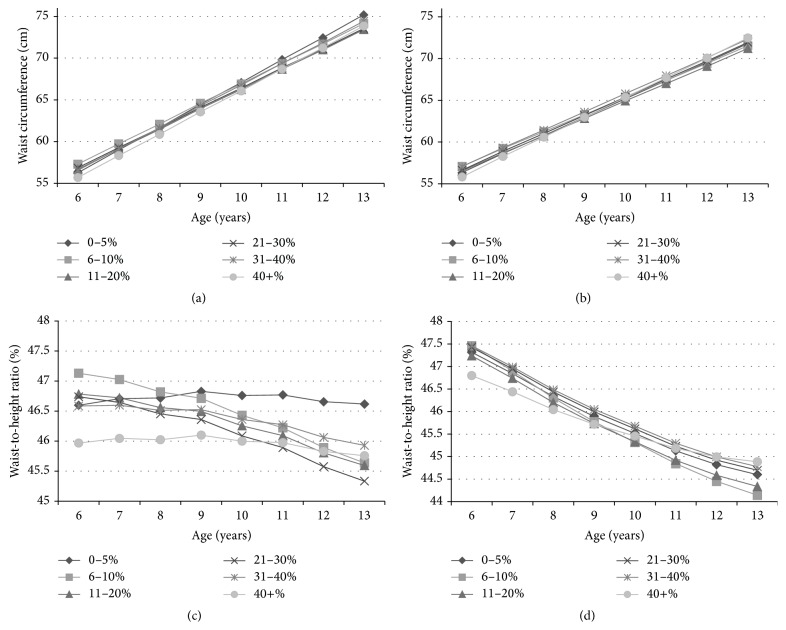
Multilevel growth curves over age, by green space category (fully adjusted): (a) boys' waist circumference; (b) girls' waist circumference; (c) boys' waist-to-height ratio; (d) girls' waist-to-height ratio.

The outcome of the linear regression models for WC can be seen in Table 2. For WC, a statistically significant interaction between green space and age was noted for boys (*p* < 0.001) and remained significant after controlling for SES (*p* < 0.001). The same interaction effect was noted for girls but was only significant after controlling for SES (*p* = 0.026), known as negative confounding [16]. Additionally, there was no significant difference between green space groups for girls, despite the overall interaction effect being significant. For boys, the interactions indicated that those in moderate green space areas (i.e., 6–30%) increased WCs, an average of 2.1 cm per year less than those in areas with little to no green space (i.e., 0–5%). This was particularly notable after age 7 (see Figure 1).

The results for WtHR can be seen in Table 3. As with WC, there was a statistically significant interaction between green space and age for boys (*p* < 0.001), which remained after controlling for SES (*p* < 0.001). The interaction effect indicated that boys who lived within an area proximal to a moderate amount of green space had on average a 0.14 slower increase in WtHRs per year than those in low green space area. The interaction effect was only noted when the boys were older (i.e., 8 years or older). There were no significant associations between green space and WtHR for girls.

## 4. Discussion

The primary aim of this study was to examine if using different measures of weight status detected different associations between green space and weight status. The results indicated that there is a statistically significant association between green space and changes in both boys' and girls' WC and between green space and changes in boys' WtHR. For both WC and WtHR, children with more green space had lower increase in average WC or WtHRs. This association only manifests, however, when the children were older.

The finding that both WC and WtHR are associated with green space reinforces previous evidence regarding the relationship between green space and BMI. Specifically, the finding that boys' WC and WtHR are negatively associated with green space indicates that children in low green space areas may be prone to additional visceral fat gain, increasing their risk of future health complications [2, 4]. This is of further importance given a recent push for measures of metabolic risk other than BMI to be considered in public health research [4].

Among boys, the results revealed that the greatest differences to the reference category (0–5% green space) by age 13 were seen in the categories with moderate amounts of green space (11–30% green space). No significant effects were noted for either WC or WtHR for those in areas of >30% green space. This nonlinear relationship between green space and children's weight status has also been noted in research using BMI as a measure of weight status [1]. One potential explanation for this finding may relate to perceived safety. For example, while some green space may be necessary to provide places for children to play in, too much green space may be perceived negatively, as it may be perceived as places which conceal criminal activity [17].

The lack of significant associations for girls were also consistent with findings from research using BMI as the measure of weight status [1]. Hypothesised mechanisms that drive green space associations are largely focused upon physical recreation [18], in which participation is known to vary substantially between boys and girls as they reach adolescence [19]. Therefore, opportunities to promote outdoor activities in greener neighbourhoods for girls warrant greater attention. Contrary to expectations, in girls the highest green space category (>40%) appeared to have the greatest average WC and WtHR by age 13, although this finding was not statistically significant. It is plausible that this unexpected result is also explained by unmeasured factors within the green space, such as the type or quality of the green space. For example, type of green space may be unimportant at younger ages when children are supervised by their parents, but it becomes more important as they develop autonomy. Independent mobility, a concept closely related to autonomy, varies significantly by gender at older ages [20]. However, the purpose of this paper was to describe the nature of associations, not to investigate potential mechanisms.

The strengths of this study include the use of longitudinal data, objective measures of weight status, and an objective measure of green space. Limitations of this study include green space only measuring quantity rather than quality, and at one point in time. The inclusion of data on green space quality may strengthen the findings for areas with high quality green space [21]. Indeed, green space quality may explain gender differences noted in this study. For example, parents of girls may view the safety of green space as paramount to relinquishing unsupervised activity, while parents of boys may be more relaxed [22]. However, given the large number of areas included in the present study, it was not feasible to collect such data.

There are a number of important future areas of research into green space influences on health. Most pressing is a greater understanding of the mediating factors which explain any relationship between green space and health. Particular focus should be on examining the causes of the gender differences noted here and in previous research on weight status [1]. While explanations such as variation in physical activity are perhaps likely to mediate relationships between green space and health, attention should also be paid to the social aspects of green space, such as safety and social cohesion.

## 5. Conclusions

Green space is associated with lower WC and lower WtHR in childhood, particularly for boys. These benefits tended to emerge after the age of 7. With green space being championed by policymakers and planners as central to healthy neighbourhoods, the gender and age-related contingencies observed for child weight status warrant further research.

## Figures and Tables

**Table 1:** Sample characteristics.

	Wave 2		Wave 3		Wave 4		Wave 5	
	Boys	Girls	Boys	Girls	Boys	Girls	Boys	Girls
Sample	2277	2187	2212	2119	2133	2036	2021	1935
Age in years (SD)	6.28 (0.45)	6.30 (0.46)	8.26 (0.44)	8.27 (0.44)	10.32 (0.47)	10.33 (0.47)	12.41 (0.49)	12.42 (0.49)
Waist circumference, cm (SD)	57.28 (5.75)	57.24 (5.92)	62.38 (7.97)	61.88 (7.60)	67.50 (9.49)	65.94 (9.19)	72.82 (10.88)	70.92 (9.81)
WtHR % (SD)	46.80 (4.02)	47.23 (4.28)	46.48 (5.13)	46.55 (5.08)	46.45 (5.79)	45.28 (5.78)	45.99 (6.30)	44.86 (5.93)
Green space 0–5%	408 (17.94%)	361 (16.54%)	384 (17.91%)	337 (16.46%)	366 (17.88%)	322 (16.43%)	344 (17.65%)	312 (16.72%)
Green space 6–10%	381 (16.75%)	371 (16.99%)	358 (16.70%)	348 (16.99%)	339 (16.56%)	342 (17.45%)	326 (16.73%)	329 (17.63%)
Green space 11–20%	678 (29.82%)	652 (29.87%)	629 (29.34%)	609 (29.74%)	598 (29.21%)	572 (29.18%)	571 (29.30%)	540 (28.94%)
Green space 21–30%	315 (13.85%)	346 (15.85%)	300 (13.99%)	326 (15.92%)	287 (14.02%)	314 (16.02%)	274 (14.06%)	300 (16.08%)
Green space 31–40%	231 (10.16%)	204 (9.34%)	225 (10.49%)	190 (9.28%)	217 (10.60%)	184 (9.39%)	205 (10.52%)	172 (9.22%)
Green space >40%	261 (11.48%)	249 (11.41%)	248 (11.57%)	238 (11.62%)	240 (11.72%)	226 (11.53%)	229 (11.75%)	213 (11.41%)
Weekly income, thousands (SD)	1.77 (1.17)	1.77 (1.20)	2.07 (1.46)	2.10 (1.51)	2.25 (1.97)	2.31 (2.05)	2.58 (2.00)	2.52 (1.69)
Indigenous or Torres Strait Islander (%)	74 (3.25%)	79 (3.61%)	59 (2.67%)	65 (3.07%)	56 (2.63%)	62 (3.05%)	57 (2.82%)	56 (2.89%)
Maternal years education (SD)	14.58 (2.47)	14.55 (2.69)	14.79 (2.46)	14.73 (2.63)	14.89 (2.50)	14.88 (2.62)	15.05 (2.48)	15.03 (2.61)
LOTE (%)	268 (11.78%)	249 (11.39%)	246 (11.12%)	220 (10.38%)	224 (10.50%)	218 (10.71%)	168 (8.31%)	159 (8.22%)

*Note*. WtHR = waist-to-height ratio; SD = standard deviation; LOTE = language other than English.

**Table 2:** The influence of green space on children's waist circumference.

	Boys (*N* = 6451)			Girls (*N* = 6108)		
	Model 1	Model 2	Model 3	Model 1	Model 2	Model 3
Fixed effects						
Age	2.53 (2.49, 2.58)^*∗∗∗*^	2.71 (2.60, 2.81)^*∗∗∗*^	2.73 (2.61, 2.85)^*∗∗∗*^	2.21 (2.16, 2.25)^*∗∗∗*^	2.24 (2.13, 2.35)^*∗∗∗*^	2.22 (2.10, 2.35)^*∗∗∗*^
Green space 0–5%						
Green space 6–10%	−0.31 (−1.46, 0.84)	1.58 (−0.20, 3.36)	2.82 (0.86, 4.78)^*∗∗*^	0.22 (−0.92, 1.35)	0.95 (−0.84, 2.74)	1.64 (−0.31, 3.60)
Green space 11–20%	−0.61 (−1.63, 0.40)	1.66 (0.09, 3.24)^*∗*^	2.30 (0.57, 4.04)^*∗∗*^	−0.43 (−1.43, 0.58)	0.16 (−1.43, 1.75)	0.86 (−0.89, 2.60)
Green space 21–30%	−0.86 (−2.06, 0.35)	2.11 (0.23, 3.98)^*∗*^	2.51 (0.47, 4.55)^*∗*^	−0.10 (−1.25, 1.05)	0.22 (−1.60, 2.05)	0.44 (−1.53, 2.42)
Green space 31–40%	−0.27 (−1.61, 1.07)	0.39 (−1.67, 2.45)	1.21 (−1.01, 3.44)	0.29 (−1.04, 1.63)	0.69 (−1.44, 2.81)	0.89 (−1.44, 3.22)
Green space >40%	−1.15 (−2.44, 0.14)	−0.11 (−2.10, 1.87)	0.09 (−2.12, 2.30)	−0.21 (−1.47, 1.05)	−1.17 (−3.16, 0.83)	−1.54 (−3.71, 0.63)
Green space *p*	0.5201	0.0775	0.0141	0.7946	0.4356	0.0892
Green space 0–5% × age						
Green space 6–10% × age		−0.21 (−0.36, −0.06)^*∗∗*^	−0.30 (−0.47, −0.12)^*∗∗*^		−0.08 (−0.23, 0.07)	−0.15 (−0.32, 0.03)
Green space 11–20% × age		−0.25 (−0.38, −0.12)^*∗∗∗*^	−0.31 (−0.47, −0.16)^*∗∗∗*^		−0.06 (−0.20, 0.07)	−0.11 (−0.27, 0.05)
Green space 21–30% × age		−0.33 (−0.49, −0.17)^*∗∗∗*^	−0.32 (−0.50, −0.14)^*∗∗*^		−0.04 (−0.19, 0.12)	−0.02 (−0.20, 0.15)
Green space 31–40% × age		−0.07 (−0.25, 0.10)	−0.15 (−0.35, 0.04)		−0.04 (−0.23, 0.14)	−0.03 (−0.24, 0.18)
Green space >40% × age		−0.11 (−0.28, 0.05)	−0.11 (−0.31, 0.09)		0.11 (−0.06, 0.28)	0.17 (−0.03, 0.37)
Green space × age *p*		0.0003	0.0004		0.3054	0.0255
Combined weekly income (thousands)			−0.14 (−0.25, −0.03)^*∗*^			−0.01 (−0.14, 0.11)
Child indigenous status			2.27 (0.25, 4.29)^*∗*^			3.18 (1.27, 5.09)^*∗∗*^
Maternal years education			−0.07 (−0.19, 0.05)			−0.25 (−0.36, −0.14)^*∗∗∗*^
LOTE			1.25 (0.30, 2.20)^*∗*^			−0.44 (−1.37, 0.49)

Random effects						
Area	2.25 (0.86, 5.89)	2.23 (0.85, 5.89)	2.29 (0.84, 6.20)	2.48 (1.07, 5.74)	2.47 (1.07, 5.74)	1.93 (0.68, 5.46)
Individual	50.29 (46.64, 54.23)	50.34 (46.69, 54.28)	46.63 (42.91, 50.67)	43.87 (40.53, 47.49)	43.87 (40.53, 47.48)	39.16 (35.87, 42.76)
Residual	21.48 (20.73, 22.26)	21.4 (20.65, 22.18)	20.31 (19.48, 21.18)	20.62 (19.88, 21.39)	20.60 (19.86, 21.37)	19.91 (19.08, 20.78)

*Note*. LOTE = language other than English. Model 1 – influence of green space and age on waist circumference; model 2 – model 1 + green space × age interaction; model 3 – model 2 + socioeconomic controls. *∗∗∗* = *p* < 0.001; *∗∗* = *p* < 0.01; *∗* = *p* < 0.05. Overall variable *p* values from Wald tests.

**Table 3:** The influence of green space on children's waist-to-height ratio.

	Boys (*N* = 6414)			Girls (*N* = 6070)		
	Model 1	Model 2	Model 3	Model 1	Model 2	Model 3
Fixed effects						
Age	−4.32 (−6.38, −2.26)^*∗∗∗*^	−4.19 (−6.25, −2.14)^*∗∗∗*^	−3.50 (−5.82, −1.18)^*∗∗*^	3.71 (1.59, 5.84)^*∗∗*^	3.69 (1.57, 5.82)^*∗∗*^	4.66 (2.23, 7.08)^*∗∗∗*^
Age^2^	0.48 (0.25, 0.70)^*∗∗∗*^	0.47 (0.25, 0.70)^*∗∗∗*^	0.41 (0.15, 0.66)^*∗∗*^	−0.47 (−0.71, −0.24)^*∗∗∗*^	−0.47 (−0.71, −0.24)^*∗∗∗*^	−0.58 (−0.85, −0.31)^*∗∗∗*^
Age^3^	−0.02 (−0.03, −0.01)^*∗∗∗*^	−0.02 (−0.03, −0.01)^*∗∗∗*^	−0.01 (−0.02, −0.01)^*∗∗*^	0.02 (0.01, 0.03)^*∗∗∗*^	0.02 (0.01, 0.03)^*∗∗∗*^	0.02 (0.01, 0.03)^*∗∗∗*^
Green space 0–5%						
Green space 6–10%	−0.31 (−1.05, 0.43)	0.97 (−0.18, 2.12)	1.80 (0.54, 3.07)^*∗∗*^	−0.11 (−0.83, 0.62)	0.41 (−0.76, 1.58)	0.05 (−1.09, 1.19)
Green space 11–20%	−0.32 (−0.98, 0.33)	1.02 (0.00, 2.04)	1.22 (0.10, 2.34)^*∗*^	−0.38 (−1.02, 0.26)	−0.34 (−1.38, 0.70)	0.14 (−1.15, 1.43)
Green space 21–30%	−0.67 (−1.44, 0.10)	1.19 (−0.02, 2.40)	1.36 (0.05, 2.67)^*∗*^	−0.15 (−0.89, 0.59)	−0.02 (−1.22, 1.17)	0.07 (−1.45, 1.60)
Green space 31–40%	−0.29 (−1.16, 0.57)	0.21 (−1.12, 1.54)	0.55 (−0.88, 1.99)	−0.08 (−0.93, 0.78)	0.01 (−1.39, 1.40)	−1.20 (−2.62, 0.22)
Green space >40%	−0.82 (−1.65, 0.01)	−0.46 (−1.75, 0.83)	−0.42 (−1.85, 1.01)	−0.32 (−1.13, 0.49)	−1.10 (−2.41, 0.20)	0.05 (−1.09, 1.19)
Green space *p*	0.4089	0.0497	0.0096	0.867	0.3198	0.2404
Green space 0–5% × age						
Green space 6–10% × age		−0.14 (−0.24, −0.04)^*∗∗*^	−0.21 (−0.33, −0.10)^*∗∗∗*^		−0.06 (−0.16, 0.04)	−0.08 (−0.20, 0.03)
Green space 11–20% × age		−0.15 (−0.23, −0.06)^*∗∗*^	−0.17 (−0.27, −0.07)^*∗∗*^		0.00 (−0.10, 0.09)	−0.02 (−0.13, 0.08)
Green space 21–30% × age		−0.21 (−0.31, −0.10)^*∗∗∗*^	−0.20 (−0.32, −0.09)^*∗∗*^		−0.01 (−0.12, 0.09)	0.00 (−0.12, 0.11)
Green space 31–40% × age		−0.06 (−0.17, 0.06)	−0.10 (−0.22, 0.03)		−0.01 (−0.13, 0.11)	0.01 (−0.13, 0.15)
Green space >40% × age		−0.04 (−0.15, 0.07)	−0.04 (−0.16, 0.09)		0.09 (−0.03, 0.20)	0.11 (−0.02, 0.25)
Green space × age *p*		0.0004	0.0003		0.275	0.1082
Combined weekly income (thousands)			−0.12 (−0.19, −0.05)^*∗∗*^			−0.05 (−0.13, 0.03)
Child indigenous status			1.83 (0.59, 3.06)^*∗∗*^			1.67 (0.46, 2.89)^*∗∗*^
Maternal years education			−0.07 (−0.14, 0.01)			−0.18 (−0.25, −0.11)^*∗∗∗*^
LOTE			0.98 (0.39, 1.57)^*∗∗*^			−0.28 (−0.88, 0.32)

Random effects						
Area	1.62 (0.91, 2.88)	1.62 (0.91, 2.88)	1.35 (0.68, 2.68)	1.02 (0.44, 2.34)	1.02 (0.44, 2.34)	0.57 (0.15, 2.14)
Individual	17.84 (16.50, 19.30)	17.87 (16.52, 19.32)	16.52 (15.15, 18.01)	17.71 (16.35, 19.19)	17.72 (16.35, 19.20)	15.72 (14.39, 17.17)
Residual	8.99 (8.68, 9.32)	8.96 (8.64, 9.29)	8.66 (8.30, 9.03)	9.12 (8.79, 9.46)	9.11 (8.78, 9.45)	8.87 (8.50, 9.26)

*Note*. LOTE = language other than English; age^2^ = age squared; age^3^ = age cubed. Model 1 – influence of green space and age on waist-to-height ratio; model 2 – model 1 + green space × age interaction; model 3 – model 2 + socioeconomic controls. *∗∗∗* = *p* < 0.001; *∗∗* = *p* < 0.01; *∗* = *p* < 0.05. Overall variable *p* values from Wald tests.
